# Sustainability through lignin valorization: recent innovations and applications driving industrial transformation

**DOI:** 10.1186/s40643-025-00929-x

**Published:** 2025-08-22

**Authors:** Poulose Sarojam Jiju, Anil Kumar Patel, Nalinakshan Sreevidya Shruthy, Saseendran Shalu, Cheng-Di Dong, Reeta Rani Singhania

**Affiliations:** 1 Institute of Aquatic Science and Technology, National Kaohsiung University of Science and Technology, Kaohsiung City, 81157 Taiwan; 2 Centre for Energy and Environmental Sustainability, Lucknow, 226 029 India; 3 Bharga Biotech and Re-Energy Solutions Private Limited, Palakkad, 679 503 India; 4 Department of Marine Environmental Engineering, National Kaohsiung University of Science and Technology, Kaohsiung City, 81157 Taiwan

**Keywords:** Lignin depolymerization, Circular economy, Bio-materials, Green chemistry, Sustainable manufacturing

## Abstract

Lignin, the second most abundant natural polymer, is emerging as a strategic feedstock for advancing a sustainable and circular bioeconomy. Traditionally underutilized byproduct of lignocellulosic biomass processing, lignin is now central to the development of bio-based chemicals, advanced polymers, and renewable fuels. This review explores recent progress in lignin valorization, focusing on emerging strategies in chemical, thermophysical, and biological depolymerization. Innovations in catalytic conversion, ionic liquid-assisted processing, and biocatalytic approaches are enabling the selective transformation of lignin into high-value products, offering viable alternatives to petroleum-derived inputs. Emerging lignin-based technologies are being adopted across key industries such as packaging, textiles, automotive, construction, and energy, enabling the development of eco-friendly and high-value product solutions. Despite its significant potential, lignin valorization is constrained by challenges including structural complexity, inefficient processing, and scalability limitations. Addressing these issues requires integrated strategies that combine technological innovation, interdisciplinary collaboration, industrial integration, and supportive policy frameworks. Through a critical assessment of the opportunities and limitations in lignin valorization and technology development, this study establishes lignin as a strategic component of next-generation biorefinery systems, advancing green material innovation and enabling resource-efficient, low-emission production in alignment with global sustainability objectives and future industrial research.

**Supplementary Information:**

The online version contains supplementary material available at 10.1186/s40643-025-00929-x.

## Graphical abstract

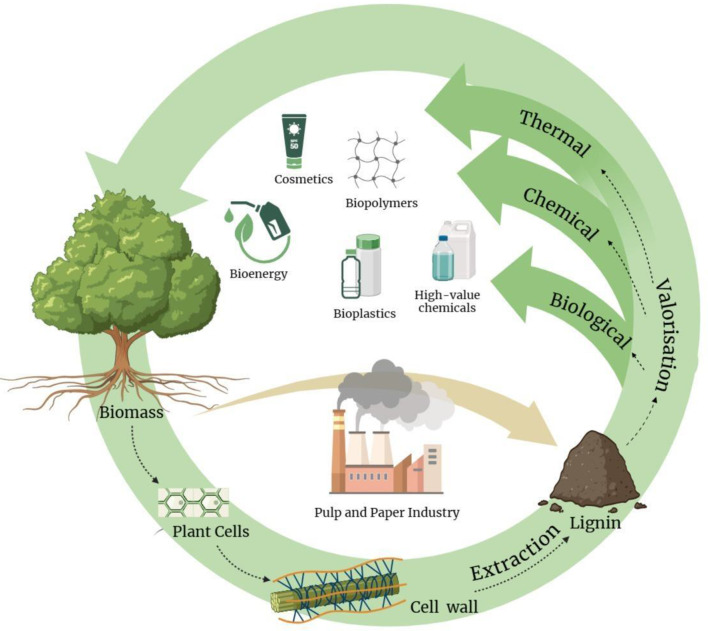

## Introduction

This is an era where industries are increasingly turning towards greener alternatives and sustainable practices, where businesses are no longer just profit-driven but purpose-driven, striving to combat climate change through bold industrial transformation. This movement is redefining industrial operations, ensuring that sustainability is a core principle to achieve net-zero emissions. This revolution lies under the concept of circular economy, where waste is no longer discarded but transformed into valuable resources. Key drivers for lignin valorization include the need to reduce greenhouse gas emissions, decrease reliance on non-renewable resources, and promote innovative material and energy solutions that align with global sustainability goals (Guo et al. 2024; Lee and Tsai 2022). Standard practices like recycling, upcycling, and reimagining byproducts can help to create a closed-loop system that minimizes environmental impact. In this context, the untapped potential of lignin could be a pioneer towards the development of lignin economy, which is unexplored due to the intricate structure in abundantly available lignocellulosic resources of the planet Earth as a lignin reservoir. The lignocellulosic biomass structure primarily consists of cellulose, accounting for 35–50%, and hemicellulose, making up 20–35%, both of which are polysaccharides. Additionally, lignin, a polyphenolic aromatic polymer, constitutes 15–30% of its composition (Sharma et al. 2023; Singhania et al. 2022). Lignin is the critical component of plant cell walls and provides structural integrity, rigidity, and protection, also contributing to the plant’s resistance against pathogens and environmental stress (Saini et al. 2023).

Lignin is a complex, amorphous heteropolymer primarily composed of three phenylpropanoid monomers: guaiacyl alcohol, *p*-coumaryl alcohol, and syringyl alcohol. For instance, lignin derived from softwood is predominantly composed of guaiacyl (G) units, accounting for 80%-90% of its structure, whereas hardwood lignin features a mix of guaiacyl (25%-50%) and syringyl (S) units (50%-70%). In contrast, lignin obtained from grasses contains a combination of guaiacyl (25%-50%), syringyl (25%-50%), and p-hydroxyphenyl (H) units (10%-25%). Conducting structural analyses and identifying the linkages within lignin are critical steps for its effective conversion into renewable fuels, materials, and chemicals (Cao et al. 2019).

A shift toward sustainable practices has led to intensified research on converting lignin into high-value biomaterials. Forest residues, agricultural byproducts, and lignin-rich industrial waste offer renewable feedstocks for creating bio-based chemicals, resins, adhesives, composites, and biodegradable plastics (Cao et al. 2019; Ramzan et al. 2023). Woody forest residues and agricultural byproducts, such as cereal straw, are abundant sources of cellulose, hemicellulose, and lignin. Lignin’s aromatic structure makes it an attractive feedstock for producing bio-based chemicals, resins, adhesives, and even biodegradable plastics, paving the way for greener alternatives in various industries. Currently, lignin is emerging as a sustainable powerhouse poised to redefine multiple industries (Ullah et al. 2022).

Among the most environmentally damaging industries today is the leather industry. Conventional leather production relies heavily on animal agriculture and toxic tanning chemicals such as chromium salts, which result in significant carbon emissions, water pollution, and hazardous waste. Additionally, the process contributes to deforestation, biodiversity loss, and raises ethical concerns about animal welfare. Even synthetic leather, often made from petroleum-based polymers like PVC and PU, poses sustainability challenges due to its non-biodegradable nature and microplastic pollution. These issues highlight the urgent need for sustainable alternatives (Sivaram and Barik 2018).

Modern industrial chemistry is transitioning from fossil-based resources to bioplastics, bio-composites, and bio-leather, with lignin playing a crucial role in sustainable polymer advancements (Otoni et al. 2021). As a versatile, low-cost material, lignin contributes to energy conservation and minimizes environmental impact (Singhania et al. 2022). The decline in fossil fuel reserves and growing environmental concerns have increased interest in renewable alternatives, with lignocellulosic biomass being a key research focus (Shankar et al. 2019; Wong et al. 2020). As industries shift from fossil-based materials to bio-based alternatives such as bioplastics, bio-composites, and bio-leather, lignin emerges as a promising, cost-effective, and environmentally friendly resource for the development of sustainable polymer technologies. Its efficient utilization contributes to a zero-waste circular bioeconomy by substituting petroleum-derived inputs with renewable lignin-based products (Alinejad et al. 2019; Ren et al. 2019). Traditionally burned for energy in pulp, paper, and biofuel industries, lignin is now being explored for high-value applications due to its abundance (15–30% of plant biomass) and structural complexity (Sun and Peng 2023; Wang et al. 2018).

The complex and diverse structure of lignin is a real challenge for valorization. However, advanced characterization techniques provide deeper insights into lignin’s structure and reactivity, facilitating the design of customized conversion pathways. Chemical depolymerization methods such as acid hydrolysis, alkaline hydrolysis, oxidative cleavage, and ionic liquid-assisted depolymerization are effective approaches used for breaking down lignin into smaller aromatic compounds. Additionally, physical methods like pyrolysis convert lignin into bio-oil, syngas, and char, making them suitable for energy and material applications. Biological approaches, including enzymatic depolymerization and microbial degradation, utilize biocatalysts to achieve the selective breakdown of lignin under mild conditions. These techniques, often used in combination, are crucial for overcoming the structural recalcitrance of lignin and optimizing its conversion efficiency (Baruah et al. 2018). However, its complex and variable structure poses processing challenges, the green chemistry and biocatalytic techniques are being developed to maximize lignin conversion (Ullah et al. 2022; Brienza et al. 2024). 

Petroleum-based materials pose environmental risks, driving the need for sustainable alternatives. Biomaterials derived from lignocellulosic biomass, particularly lignin, offer a promising solution. Although traditionally underutilized, recent advancements have unlocked lignin’s potential in sustainable product development (Rial 2024). These innovations have improved its extraction and conversion, enabling applications in biodegradable plastics, composite materials, and more. The growing field of lignin valorization integrates green chemistry, biotechnology, and nanotechnology to enhance the performance of lignin-based materials, increasing their commercial viability. This shift towards renewable, plant-based resources is poised to revolutionize industries such as packaging, automotive, and construction, fostering material independence from fossil resources and contributing to a circular economy (Moshood et al. 2022).

This review explores the evolving role of lignin as a value-added feedstock within lignocellulosic biorefineries, emphasizing its potential in the production of advanced materials. Traditionally regarded as a recalcitrant byproduct, lignin is now recognized as a critical component in biorefinery schemes, offering a renewable source for industrial applications. Recent progress in thermochemical and catalytic depolymerization, as well as enzymatic and microbial modification strategies, has facilitated its conversion into bio-based chemicals, polymers, structural composites, and leather-like materials. Despite these developments, the transition to industrial-scale implementation remains constrained by challenges such as intrinsic structural heterogeneity, low process selectivity, and inconsistent material performance. Strategic lignin valorization within integrated biorefinery frameworks is imperative for decreasing petrochemical dependency and enabling a circular bioeconomy aligned with global sustainability objectives.

## Current techniques of lignin valorization

The effective utilization of lignin requires the implementation of efficient and targeted valorization strategies. Understanding the strengths and limitations of each approach is crucial for determining their suitability across various lignin sources and industrial contexts. Table 1, summarizes the range of lignin valorization techniques, highlighting their respective benefits and associated challenges.

**Table 1:** Advantages and disadvantages of lignin valorization methods

valorization techniques	Feedstock	Advantages	Disadvantages	Biomass source and lignin recovery		References
				Biomass (Lignin%)	Extraction yield (%)	
Alkaline hydrolysis	Agricultural residuals: Rice straw, wheat straw, sunflower stalk	Operates under lower temperatures	Requires an expensive catalyst and the formation of inhibitors	De-oiled Jatropha waste (40–50%)	93.4%	Novakovic et al. 2021; Oruganti et al. 2024
Acid hydrolysis (dilute)	Wheat straw	Acid recycling is not required	High temperature and pressure are needed	Corn stover (17–21%)	75.7%	Gill et al. 2021; Sheng et al. 2022; Tu and Hallett 2019
Acid hydrolysis (concentrated)	Wheat straw and bagasse	Operates under mild temperatures	Acid recovery is expensive and requires corrosion-free equipment	Sugar cane (23.7%)	91%	Ávila and Rosana 2022; Momayez et al. 2022
Oxidative cleavage	Wheat straw, bagasse, and poplar sawdust	Effective lignin degradation	Expensive reagent requirement	*Toonna sinensis* branches (13%)	77%	Lin et al. 2024; Wei et al. 2021
Ionic liquid-assisted depolymerization	Wheat straw, bagasse and peanut	Sufficient dissolution	Expensive ionic liquids are required in higher amounts	*Miscanthus x giganteus* (17–25%)	82%	Radhakrishnan et al. 2021; Taylor et al. 2024; Wang and Eika 2020
Pyrolysis	Various lignin sources, such as dry impregnated lignin and agricultural residues	Simple and less-expensive	Higher temperature is required for the initiation of decomposition, which results in reduced yield of bio-oil	Tobacco waste (18.6%)	85%	Lu and Gu 2022; Tan et al. 2024; Wang et al. 2023
Microbial depolymerization by fungi	Rice straw, wheat straw, and softwood	Moderate conditions and minimal energy are sufficient, cost-effective	A wide sterile area, low hydrolysis rate, and more time are required	Sugarcane baggase (20–30%)	87.6%	Dong et al. 2013; Giri and Sharma 2020; Hermosilla et al. 2018
Microbial depolymerization by bacteria	Rice straw, wheat straw, and softwood	Moderate conditions are sufficient, cost-effective		Paper mill waste	89%	Gu et al. 2024; Hemati et al. 2023; Kumar et al. 2022; Nurika et al. 2022
Enzymatic depolymerization	Rice straw, wheat straw, and softwood	Moderate conditions and minimal energy are sufficient		Paper mill waste	90%	Kumar et al. 2022; Weiss et al. 2020; Zhang et al. 2021

### Chemical depolymerization

Chemical depolymerization breaks down lignin’s complex polymer structure into smaller molecules, enabling its conversion into valuable chemicals and fuels. Compared to high-temperature methods like pyrolysis or the low efficiency of biological processes, chemical treatments offer better reaction control and higher product selectivity, enhancing lignin’s potential for renewable fuel and chemical production (Klinger et al. 2020). The process can be categorized into several methods based on the chemicals used such as acidolysis, alkaline hydrolysis, oxidative cleavage, ionic liquid extraction, catalytic or enzymatic depolymerization. Chemical depolymerization is often combined with thermal methods in a process called thermochemical depolymerization (Roy et al. 2022).

## Alkaline hydrolysis

Alkaline depolymerization uses strong bases to break lignin into smaller, valuable compounds, mainly phenolic monomers and oligomers. This method is particularly effective due to its ability to suppress repolymerization and char formation, which are some of the critical problems in lignin processing (Zhou et al. 2022). Base-catalysed depolymerization (BCD) typically occurs under harsh conditions, often at temperatures above 300 °C and high pressures (over 200 bar). The primary mechanism involves the cleavage of aryl-alkyl bonds, particularly the β-O-4 ether linkages. The cleavage results in the formation of smaller phenolic compounds, such as guaiacol, catechol, and vanillin, which can be further processed or utilized. Strong alkaline agents such as sodium hydroxide (NaOH), potassium hydroxide (KOH) are widely used for the process. Calcium hydroxide (Ca (OH)₂), although milder in effect, is still a valuable option due to its lower cost and accessibility (Rajesh et al. 2019). The use of base catalysts in BCD reactions can significantly enhance the yield of phenolic monomers from lignin. Recent advancements have expanded the possibilities of base depolymerization through NaOH-incorporated choline chloride (ChCl): urea mixtures, which facilitate lignin depolymerization at atmospheric pressure (1 atm). The use of 7.5% NaOH in an aqueous ChCl: urea solution, attained an aromatic monomer yield of 23.06 mg/g (Biswas et al. 2021; Ong et al. 2023). The highest lignin recovery for alkaline hydrolysis was reported as 93.4% using NaOH treatment of de-oiled *Jatropha* waste, highlighting its efficiency under optimized alkaline conditions (Oruganti et al. 2024).

Acid-precipitated lignin (APL), obtained by acidifying the alkaline hydrolysate of lignin, is rich in phenolic and carboxylic groups, enhancing its suitability for a wide range of applications. Its aromatic structure and high reactivity make it an excellent candidate for producing bio-based adhesives and coatings. APL is also a sustainable raw material for manufacturing phenolic resins, polyurethane precursors, and nanolignin dispersions. Moreover, it holds potential in environmental and agricultural sectors, serving as an effective adsorbent for heavy metal removal in water treatment and as a component in slow-release fertilizers. Its renewable and biodegradable characteristics make APL a promising substitute for petroleum-based materials in sustainable chemistry and material innovation (Akshita et al. 2024).

Alkaline hydrolysis offers several advantages in lignin depolymerization, notably its high selectivity in producing valuable phenolic compounds used in pharmaceuticals, agrochemicals, and as biofuel precursors. This method is also considered more environmentally friendly, often generating fewer toxic byproducts compared to acidic methods (Ahmad et al. 2018). However, alkaline hydrolysis presents challenges, including the corrosive nature of strong bases, which require specialized reactor materials. Furthermore, the recovery and recycling of bases increase operational costs, particularly at a large scale. A major limitation is that alkaline hydrolysis can produce inhibitory compounds that disrupt downstream processes like fermentation, complicating the integration of alkaline hydrolysis into biorefinery systems.

## Acid hydrolysis

Acid hydrolysis is a significant method for the valorisation of lignin, which involves the use of acidic conditions to break down lignin into smaller, more valuable compounds, primarily phenolic monomers and oligomers. Various acids are employed for lignin hydrolysis, with mineral acids such as hydrochloric and sulfuric acid being particularly effective due to their strong protonating capabilities. Organic acids like formic and acetic acid are also used, often in combination with alcohols, to enhance selectivity toward targeted products (Zhou et al. 2022). The catalytic effect of HCl, H_2_SO_4_, H_3_PO_4_, and formic acid (FA) was studied using mild microwave-assisted heating at 160 °C. Among these, FA and H_2_SO_4_ proved effective catalysts, producing lower molecular weight compounds with minimal solid residue, whereas H_3_PO_4_ promoted undesirable repolymerization. The depolymerization of oxidized lignin under mild conditions in aqueous FA yielded more than 60 wt% of low-molecular-mass aromatics. A diverse range of acids, including mineral acids, Lewis acids, zeolites, organic acids, and acidic ionic liquids has proven effective for lignin conversion. In particular, Lewis acids (such as metal chlorides and metal triflates) exhibit catalytic activity by interacting with water or alcohol, transforming into their corresponding Brønsted acids and thereby facilitating the cleavage of lignin ether bonds (Rajesh et al. 2019).

The process of acidolysis typically involves the cleavage of the β-O-4 ether bonds, which are the most abundant linkages in lignin. In a highly acidic environment created by strong acids like hydrochloric, sulfuric, or formic acid, the oxygen in the ether bond becomes protonated, which increases its susceptibility to nucleophilic attack. Water molecules or alcohols act as nucleophiles, attacking the protonated ether bond, leading to the cleavage of the bond. The cleavage results in the formation of smaller phenolic compounds, such as guaiacol, catechol, and syringol. Several factors influence the efficiency of acid hydrolysis, including acid concentration, temperature, reaction time, and pressure.

Higher concentrations of acid can enhance the reaction rate but may also lead to the degradation of valuable products if not carefully managed. Elevated temperatures, typically between 150 °C and 400 °C, can accelerate the hydrolysis process at optimum duration, but excessive temperatures may cause further decomposition of the products. In certain cases, high pressure is applied to maintain the liquid phase, enhancing reactant solubility and improving yield (Žula et al. 2023). Under dilute acid hydrolysis, the maximum lignin yield of 75.7% was obtained from corn stover treated with dilute acetic acid, indicating its potential despite requiring elevated temperature and pressure (Gill et al. 2021). A significant lignin yield of 91% was achieved using concentrated acetic acid on sugarcane bagasse, demonstrating the effectiveness of concentrated acid hydrolysis under mild temperatures (Avila and Rosana 2022). The choice of acid and conditions provides several advantages to acid hydrolysis, including high selectivity for valuable phenolic compounds used in pharmaceuticals, agrochemicals, and biofuel precursors.

## Oxidative cleavage

Oxidative depolymerization is a promising strategy for converting lignin into valuable phenolic compounds through the use of oxidizing agents. This process takes advantage of the abundant hydroxyl groups in lignin, enabling selective cleavage of specific bonds while preserving its aromatic structure (Zhou et al. 2022). Depending on the pH acidic, neutral, or alkaline the reaction conditions significantly influence the types and yields of products formed (Li and Takkellapati 2018). The mechanism generally involves electron transfer or hydrogen atom abstraction, leading to reactions such as aromatic ring hydroxylation, phenol and benzylic oxidation, demethylation, and in some cases, ring-opening (Ahmad et al. 2021). Common oxidants employed include hydrogen peroxide, nitrobenzene, oxygen, and metal oxides, which facilitate efficient depolymerization while maintaining the aromatic nature of the resulting compounds (Figueiredo et al. 2018). This approach is particularly favorable for producing aromatic aldehydes and acids like vanillin, syringaldehyde, vanillic acid, and syringic acid. Kraft lignin, with its high C–C bond content, especially in linkages like 5–5′ and α–5′, is especially suitable for this process. Moreover, the growing interest in oxidative depolymerization stems from its potential to operate under mild, cost-effective conditions, making it an attractive route for lignin valorization (Li and Takkellapati 2018).

The process efficiency, product yield, and distribution depend heavily on variables like pH and oxidant choice. Hydrogen peroxide has been studied under both acidic and alkaline conditions. Precipitated hardwood lignin can achieve similar depolymerization levels (around 98 wt%) at lower temperatures (80–90 °C) under alkaline conditions, but requires higher temperatures (130–160 °C) under acidic conditions. The oxidative cleavage method yielded a maximum of 77% lignin from *Toona sinensis* branches when treated with H₂O₂ and acetic acid, reflecting good degradation efficiency under oxidizing conditions (Lin et al. 2024). This process yields significant amounts of formic, oxalic, and acetic acids, with only minimal aromatic aldehyde production, indicating that hydrogen peroxide may not be ideal for targeting aldehydes specifically. In contrast, nitrobenzene is more effective in generating aromatic aldehydes like vanillin, syringaldehyde, and p-hydroxybenzaldehyde, as well as their related acids (e.g., vanillic acid and syringic acid), with optimal yields achieved at around 170 °C and a 2.5-hour residence time. However, metallic oxides and transition metals are sometimes preferred as safer alternative oxidants due to the carcinogenic nature of nitrobenzene (Figueiredo et al. 2018). This study highlights the potential of using safer oxidants to improve the sustainability of oxidative depolymerization and emphasizes that a deeper understanding of its mechanisms and optimal conditions can significantly advance lignin valorization for sustainable chemical production (Vangeel et al. 2018).

## Ionic liquid-assisted depolymerization

Ionic liquids (ILs), made of organic cations and inorganic or organic anions, stay liquid at or below 100 °C and are useful in lignin depolymerization. Despite frequent concerns regarding its environmental toxicity, ionic liquids (ILs) demonstrate exceptional chemical reactivity in the degradation and transformation of lignin (Ji and Lv 2020). Their ability to achieve high extraction efficiency while preserving the structural integrity of lignin makes them highly favorable for the production of aromatic feedstocks. Additionally, the ionic nature of ILs results in minimal vapor pressure, reducing the release of volatile organic compounds (VOCs) (Zhu et al. 2018).

Developing efficient lignin depolymerization strategies requires a comprehensive understanding of IL properties and the roles of different cations and anions. Alkylsulfonate anions are particularly effective for reducing the polydispersity of lignin and molecular weight, while lactates, acetates, chlorides, and phosphates enhance reactivity. Targeted cleavage of β-O-4 linkages in lignin for guaiacol production has shown that 1-hexyl-3-methylimidazolium chloride ([Hmim]Cl) provides the highest yield, followed by 1-butyl-3-methylimidazolium hydrogen sulfate ([Bmim][HSO4]), 1-hexyl-3-methylimidazolium bromide ([Hmim]Br), 1-hexyl-3-methylimidazolium hydrogen sulfate ([Hmim][HSO4]), and 1-hexyl-3-methylimidazolium tetrafluoroborate ([Hmim][BF4]). Weakly basic anions like tetrafluoroborate ([BF4]⁻) and trifluoromethanesulfonate ([CF3SO3]⁻) promote dealkylation, while moderately basic anions like chloride (Cl⁻) and bromide (Br⁻) help inhibit this reaction in the cleavage of ether bonds catalyzed by Brønsted acids. Several studies highlight the effectiveness of ionic liquids (ILs) in enzymatic lignin depolymerization by enhancing lignin solubility and increasing the yield of phenolic compounds (Liu et al. 2024). The combination of 1-ethyl-3-methylimidazolium acetate ([Emim]OAc) with laccase led to significant cleavage of β-O-4 bonds in lignin, producing valuable aromatic compounds (Quesada-Salas et al. 2023). ILs combined with a lignin-degrading enzyme cocktail not only solubilize lignin but also promote higher yields of low-molecular-weight phenolics. The selection of ILs is critical in optimizing lignin solubility and enzyme efficiency, making it essential to tailor solvent conditions for maximum lignin valorization (Zhou et al. 2022; Kumar et al. 2023). A lignin yield of 82% was recorded from *Miscanthus x giganteus* using the ionic liquid [TEA][HSO₄], indicating effective dissolution with this advanced technique (Wang and Eika 2020).

However, the high cost of ILs limits their large-scale application in lignin depolymerization, emphasizing the need for efficient recycling and separation processes. Separation of lignin-derived products from ILs is complex due to strong π-π interactions between aromatic lignin and ILs. Advanced analytical techniques like UV-visible spectroscopy, infrared spectroscopy, mass spectrometry, light scattering, and nuclear magnetic resonance (NMR) are used to analyse lignin products dissolved in ILs. Application of ILs in lignin depolymerization should address scalability, toxicity, and life cycle impacts (Weldemhret et al. 2020).

Chemical depolymerization offers a highly selective and efficient method for recovering original monomers such as ethylene, propylene, and terephthalic acid, enabling their reuse without quality loss. It has shown success in converting polymers like PET, polystyrene, polyamides, and lignin-based biopolymers into valuable feedstocks and high-value chemicals such as vanillin, catechol and guaiacol. By supporting closed-loop recycling, it reduces reliance on fossil resources, minimizes landfill waste, lowers CO₂ emissions, and contributes significantly to sustainable material recovery (Qin and Xi 2024).

### Thermophysical depolymerization

Thermophysical depolymerization of lignin refers to the breakdown of its complex polymeric structure through high-temperature processes that rely on physical and thermal mechanisms rather than chemical catalysts.

## Pyrolysis

Pyrolysis, a thermal degradation process conducted in an oxygen-free environment (Sharma and Kumar 2020). The classification of pyrolysis depends on the operating temperature and duration, which typically includes: (1) primary pyrolysis, occurring between 200 and 400 °C, and (2) secondary pyrolysis, conducted at temperatures above 400 °C. The end products of pyrolysis are highly influenced by the specific parameters applied during the process (Fahmy et al. 2020).

It can be further divided into slow pyrolysis and fast pyrolysis. In slow pyrolysis, the process typically takes over 30 min with a heating rate under 100 °C/s, resulting in a bio-oil yield around 30%. Prolonged residence times and lower heating rates reduce bio-oil yields and increase syngas production due to secondary cracking reactions. Fast pyrolysis is a highly efficient technology for converting biomass into bio-oil, characterized by rapid heating rates that accelerate bond cleavage and lead to lignin decomposition into volatile compounds. The process unfolds in three stages: moisture release, devolatilization, and breakdown of crosslinked structures (Dhyani and Bhaskar 2018). Recent studies have indicated that pyrolysis efficiency can be enhanced by using catalysts, including transition, noble, and inorganic metals as well as zeolites. Zeolites and metal compounds, including metal chlorides, have been widely utilized as catalysts in lignin pyrolysis. These metal catalysts are noted for their ability to limit the formation of undesirable chemicals, ultimately enhancing the yield of valuable pyrolytic lignin byproducts (Bi et al. 2018). Among thermal processes, pyrolysis of tobacco waste resulted in 85% yield, though it required high temperatures to initiate decomposition and bio-oil production (Tan et al. 2024).

Lignin acts as a natural reservoir of phenolic compounds, with its classification into guaiacyl (G), syringyl (S), p-hydroxyphenyl (H), or catechyl (C) types depending on the specific phenolic units in its polymeric structure. Selective removal of methoxy groups (-OCH₃) from lignin simplifies and homogenizes its structure, which not only enhances the yield of desired phenolic products, particularly H-type compounds, but also minimizes side reactions that lead to the formation of light gases. This structural refinement contributes to improved efficiency and selectivity in the lignin depolymerization process (Lu and Gu 2022).

## Biological depolymerization

The efficient utilization of lignin hinges on its depolymerization, a process facilitated by a variety of lignin-degrading enzymes and microbial metabolic systems. This biological process not only offers a cleaner and more efficient alternative to traditional chemical methods but also aligns with the principles of green chemistry and circular economy.

### Microbial depolymerization

Microbial depolymerization of lignin represents a transformative approach in the quest for sustainable biomass utilization and the production of value-added bioproducts. Lignin, a complex and recalcitrant biopolymer, poses significant challenges for its degradation and conversion due to its intricate structure and chemical stability (Andlar et al. 2018). However, certain microorganisms, particularly bacteria and fungi, have evolved specialized enzymatic systems capable of breaking down lignin into smaller, more manageable aromatic compounds. As the demand for sustainable solutions intensifies, understanding and optimizing microbial lignin depolymerization will be crucial for advancing biotechnological strategies that contribute to environmental sustainability and resource efficiency.

### Depolymerization by fungi

Fungi are particularly effective at degrading lignin due to their ability to produce a wide range of enzymes (Andlar et al. 2018). Based on degradation mechanism, lignin-degrading fungi are categorized into three main types: white-rot, brown-rot, and soft-rot fungi. In these, only white-rot fungi are capable of fully breaking down lignin into CO₂ and H₂O.

Lignin degradation by white-rot fungi primarily involves basidiomycetes, with a few ascomycetes’ species also capable of this function. These fungi secrete extracellular oxidases and peroxidases, which catalyze oxidative reactions essential for lignin breakdown, such as the cleavage of carbon–carbon bonds and ether linkages, as well as the removal of side chains and aromatic rings (Wood Microbiology 2020). *Phanerochaete chrysosporium* is often cited as a model white-rot fungus for lignin degradation and has been extensively applied in the biological pretreatment of lignocellulosic biomass produce laccase and peroxidases, which contribute to lignin oxidation and decomposition. For instance, lignin from oak wood was converted to ethanol via fermentation using *Phlebia sp*. MG-60, in which manganese peroxidase (MnP) and laccase were detected in the culture (Bautista-Zamudio et al. 2023). Brown-rot fungi associated with softwoods, comprise about 7% of wood-degrading basidiomycetes and efficiently hydrolyze cellulose and hemicellulose but only partially oxidize lignin. In contrast, soft-rot fungi, including *Aspergillus niger* and *Penicillium chrysogenum*, have been found to degrade lignin in woods like pine. Additionally, some soft-rot fungi degrade vanillic acid and phenolic compounds. Though the lignin-degrading enzymes of fungi are less well understood, they are believed to modify rather than fully mineralize lignin (Schilling et al. 2021). The highest lignin yield via fungal treatment was 87.6%, achieved from sugarcane bagasse using *Lentinula edodes* LE16, under moderate and eco-friendly conditions (Dong et al. 2013).

### Depolymerization by bacteria

Microbial depolymerization of lignin using bacteria is an emerging field that holds significant promise for the sustainable conversion of lignocellulosic biomass into valuable bioproducts. Lignin-degrading bacteria have been isolated from various environments, including soil, decaying wood, wastewater treatment plants, and the guts of animals (Xu et al. 2018). Although bacterial lignin degradation is generally less efficient than that of fungi, bacteria exhibit greater environmental resilience. Recent research has identified Actinobacteria, Proteobacteria, and Firmicutes as key groups of lignin-degrading bacteria. These bacteria secrete oxidative enzymes that facilitate lignin breakdown in the presence of oxygen (Grgas et al. 2023).

Several bacterial species, including *Pseudomonas putida*, *Rhodococcus jostii*, *Bacillus subtilis*, and *Streptomyces griseus*, are known for their lignin-degrading capabilities through the production of oxidative enzymes such as laccases, lignin peroxidases, and manganese peroxidases. These enzymes cleave the complex bonds in lignin, producing smaller aromatic compounds that can be further metabolized into biofuels, bioplastics, and other value-added products (Kamimura et al. 2019; Weng et al. 2021). Factors like pH, temperature, and oxygen availability influence degradation efficiency. Notably, species such as Arthrobacter and Clostridium thrive in extreme or anaerobic conditions, expanding the applicability of bacterial lignin valorization. The metabolic versatility of bacteria underscores their potential in sustainable lignocellulosic biomass conversion (Weng et al. 2021). Using *Bacillus cereus* on paper mill waste, bacterial depolymerization achieved a notable 89% lignin recovery, emphasizing its cost-effectiveness and environmental compatibility (Gu et al. 2024).

The potential applications of bacterial lignin degradation extend beyond bioremediation and waste management; they also encompass the development of sustainable practices in biomass utilization, contributing to a circular economy.

### Enzymatic depolymerization

Lignin peroxidases (LiPs) are a class of enzymes that play a significant role in lignin degradation. Secreted primarily by white-rot fungi, such as *Phanerochaete chrysosporium*, LiPs utilize hydrogen peroxide to oxidize lignin, leading to the formation of free radicals that can further react with lignin structures. Manganese peroxidases (MnPs), also produced by white-rot fungi, are essential for the degradation of lignin as they catalyze the oxidation of manganese ions, which in turn oxidize phenolic compounds in lignin. Versatile peroxidases (VPs) effectively depolymerize lignin due to their unique ability to oxidize its complex structure. These enzymes, found in species such as *Bjerkandera adusta*, have garnered attention for their broad substrate specificity and potential applications in bioremediation and bioconversion processes (Gałązka et al. 2025). Enzymatic treatment with laccase on paper mill waste led to a high lignin recovery of 90%, showcasing the potential of enzyme-based approaches under mild reaction conditions (Kumar et al. 2022).

The co-expression of multiple lignin-degrading enzymes in microbial hosts has been explored to create synergistic effects that enhance lignin breakdown. The integration of these enzymatic systems into biotechnological processes not only promotes the sustainable utilization of lignin but also contributes to the development of eco-friendly methods for producing biofuels, bioplastics, and other high-value chemicals. As research continues to elucidate the mechanisms and efficiencies of these enzymes, the prospects for their application in industrial lignin valorization become increasingly promising, paving the way for innovative solutions in biomass conversion and waste management (Weng et al. 2021).

### Smart solutions from lignin: applications driving the bioeconomy

The applications of lignin valorization have gained significant attention due to its potential to produce a wide range of valuable products. These products span multiple industries, including biofuels, chemicals, materials, and more, showcasing lignin’s versatile role in advancing sustainable solutions. Table 2 summarizes various lignin valorization methods, highlighting the diverse products derived from these processes.

**Table 2:** Lignin valorization technique for value-added products with application

Lignin valorization Technique	Products/Chemicals	Application	References
Reductive conversion	Aromatic phenols, Alcohols, Aldehydes, Acids and Alkyl phenols	Bio-based chemicals, additives, precursors for fragrances, vanillin, solvents, bio-based precursors, Feedstock for chemicals, biodegradable plastics	Cao et al. 2019; Grossman and Vermerris 2019
Catalytic halogenation	Benzene, Toluene, Xylene	Bio-oil and fuel production	Mukarakate et al. 2022
Hydrolytic depolymerization	Bio based polyurethenes (PU)	Bio-based foams, coatings, adhesives	Ma et al. 2023
Catalytic depolymerization	Phenolic resins	Used in different coatings, composites, adhesives, insulations, lamination, wood bonding, in molded parts, and as plywood adhesives	Solt et al. 2018
Oxidative conversion	Vanillin	Flavoring agent, used in perfumes and cosmetics, in pharmaceuticals	Wang et al. 2019a, b
Oxidative conversion	Syringaldehyde	Additives in polymers, food preservation	Wang et al. 2019a, b
Fast pyrolysis	Bio-oil	Fuel for combustion engines, feedstock for chemicals, soil fumigant, additive for asphalt and adhesives	Puziy et al. 2018; Yuan et al. 2022
Pyrolysis	Syngas	Fuel for heat and electricity, feedstock for Fischer-Tropsch synthesis (produces liquid hydrocarbons)	Puziy et al. 2018; Yuan et al. 2022
Physical or chemical activation of lignin	Activated carbon	Water and air filtration, gas purification, medicine (e.g., toxin adsorption), energy storage (supercapacitors)	Puziy et al. 2018; Yuan et al. 2022
Electrospinning	Carbon fibers	Lightweight composites in aerospace, automotive parts, sports	Poursorkhabi et al. 2020; Puziy et al. 2018
Electro chemical methods and reductive fractionation	3D printing resin (Anionic surfactant), Lignin nano tubes, Hydrogels	Biodegradable filaments, smart packaging, tissue engineering, drug delivery, wound healing, controlled moisture release, food packaging, structural composites, nanocomposites	Jindal et al. 2023; da Cruz et al. 2022
Electrospinning	Scaffolds	Tissue regeneration, bone repair, cell growth support, drug encapsulation, water filtration, agricultural applications	Ali et al. 2024a
Solvothermal techniques	Li-ion, Na-ion batteries, Supercapacitors	Electrodes, energy storage, charge storage, electrode materials	Culebras et al. 2019
Photocatalytic and electrochemical conversion	Solar cells	Energy conversion, photovoltaic materials	Sultana et al. 2024
Catalytic depolymerization, chemical modification	Bitumen, Cement additive	Soil conditioner, Fire retardant	Cao et al. 2019; Puziy et al. 2018
Chemical depolymerization	Dispersant	In fertilizers and pesticides (controlled release agent, sequestering agent)	Cao et al. 2019; Puziy et al. 2018
Biorefinery approaches and thermochemical method	Soil conditioner, Fertilizers and pesticides	Controlled release agent, sequestering agent, material absorbing soil contaminants	Brienza et al. 2024

### Reinventing polymers: the rise of lignin in green material innovation

Synthetic plastics have driven significant development, but their non-biodegradability poses serious environmental challenges. In contrast, Kraft lignin has been widely used as a filler in polymer composites to improve adsorption, structural, and morphological properties. Due to variability in lignin types, their applications differ based on specific properties (de Sousa et al. 2019). The Central Pollution Control Board of India reports that in 2019-20 alone, plastic waste generation was estimated at approximately 3.47 million tons per year (Sharma et al. 2024). Lignin has emerged as a valuable component in the field of sustainable polymer and material development and can be used directly to produce a variety of polymers, including polyesters, polyurethanes, and resins, offering an eco-friendly alternative to petroleum-based sources. Raw lignin, in its unmodified form, has been integrated directly into materials to enhance their properties. It shows promise in plastics as a cost-effective additive, serving as an antioxidant, UV protection agent, flame retardant, and reinforcement filler (Mariana et al. 2021). Despite its vast potential, lignin can only be mixed in limited quantities with plastics due to compatibility issues. Therefore, developing modified lignin or lignin-based polymers appears to be a logical first step toward wider use of this eco-friendly biopolymer across industries. These polymers are either synthesized by functionalizing the hydroxyl groups in lignin’s structure or by incorporating lignin into blends, copolymers, and composites. Its complex, polyaromatic structure provides significant mechanical strength and resilience, making it suitable for high-performance applications (Zhang et al. 2024). Lignin’s inherent polarity from its hydroxyl groups, promotes compatibility with polar polymers but limits integration with nonpolar materials, necessitating compatibilizers to improve interfacial bonding. Research has explored the application of lignin in producing lignin-based thermoplastic and thermoset composites, demonstrating that these composites exhibit promising biodegradability (Sharma and Kumar 2020). For the production of sustainable materials, such as bioplastics, blends, or bio-composites, lignin has been used as a binding matrix for natural fibers (Hilares et al. 2020).

Lignin has been widely explored for its potential to improve the performance of various biopolymer blends. A study by Kovalcik et al. demonstrated that incorporating lignin into polylactic acid (PLA) composites not only enhanced mechanical strength but also increased thermal stability (Kovalcik et al. 2015). This improvement was attributed to hydrogen bonding between the functional groups of lignin and PLA, resulting in better interfacial compatibility. Likewise, Kai et al. showed that the addition of kraft lignin to polyhydroxybutyrate (PHB) significantly boosted tensile strength from 1.81 MPa to 3.13 MPa and increased elongation at break from 15 to 55%, confirming lignin’s reinforcing capabilities. Lignin/PHB nanofibers exhibiting excellent biocompatibility and biodegradability have been developed, highlighting their potential for significant biomedical applications (Kai et al. 2019).

Nofar et al. investigated lignin’s transformative role in bio-based polyamide blends has garnered attention, with its incorporation yielding significant enhancements in thermal and mechanical properties, paving the way for innovative applications in dynamic industries like automotive and sustainable packaging (Nofar and Park 2014). In the context of flame retardancy, Chung et al. found that addition of lignin to PLA emerged as a game-changer, enhancing fire resistance and opening doors to safer, eco-friendly materials for use in high-risk, safety-critical applications (Chung et al. 2013). In addition, Yang et al. pointed out that lignin can enhance the gas barrier properties of PLA-based packaging, thereby maintaining biodegradability while elevating functional performance (Yang et al. 2016). Zhang et al. highlighted lignin’s contribution to imparting antioxidant and antimicrobial qualities to biopolymer composites, which is especially valuable in biomedical applications where microbial resistance is important (Zhang et al. 2015).

Wang et al. and Liu et al. both explored the development of polyurethane foams using lignin as a sustainable alternative to petroleum-based polyols. Wang et al. formulated a highly elastic thermosetting polyurethane foam by partially replacing conventional polyols with alkali lignin modified using polyethylene glycol (PEG2000), which was then reacted with hexamethylene diisocyanate (HDI). This modification significantly improved the flexibility of the foam, achieving an elastic recovery rate exceeding 93% when 50% of the polyols were substituted with ALPEG2000, demonstrating excellent elastic properties (Wang et al. 2019). Similarly, Liu et al. synthesized thermosetting rigid polyurethane foam (RPF) by incorporating lignin oligomers extracted from poplar via catalytic upstream biorefining (CUB) as a polyol substitute. Their study emphasized that the structural features of the lignin, particularly hydroxyl group content, along with the composition of the CUB-derived slurry and the lignin oligomer proportion, critically influenced the foam’s morphology, compressive strength, and bulk density (Liu et al. 2024). Together, these studies underscore the potential of lignin-based polyols in tailoring the mechanical and structural properties of both elastic and rigid polyurethane foams (Wang et al. 2019; Liu et al. 2024).

Shen et al. incorporated catechol-functionalized lignin as both a reinforcing agent and cross-linker to develop composite materials with polyvinyl alcohol (PVA) using various fabrication techniques, including casting–drying, freeze–thaw cycles, and freeze–salt treatment. The inclusion of catechol lignin notably enhanced the mechanical performance of the PVA films, leading to 2.1–4.6 fold increases in tensile strength, elongation at break, and Young’s modulus. Additionally, the modified films exhibited improved UV shielding, fluorescence, thermal stability, and water resistance properties (Shen et al. 2023).

Feng et al. developed a robust and long-lasting conductive lignin-based nanocomposite organogel using a binary solvent mixture of dimethyl sulfoxide and water. In this system, alkali lignin nanoparticles served as physical cross-linkers and reinforcing fillers, effectively dispersing external stress and delaying the gel’s structural failure. This dual functionality significantly enhanced the mechanical strength of the resulting organogel (Feng et al. 2022).

Carbon fibers (CFs) are highly valued for their lightweight nature, superior tensile strength, high specific modulus, low electrical resistivity, and excellent torsional resistance, often outperforming steel in mechanical properties at a significantly lower weight (Karunarathna and Smith 2020). Over the past decade, research into lignin-based CFs has shown significant commercial potential (Souto et al. 2018). Lignin has been identified as a valuable component in battery electrode production due to its cost-effectiveness and high yields during pyrolysis. Thermochemical modification of lignin can result in carbon fiber precursors with yields of approximately 30–35%, depending on lignin purity and molecular weight (Ali et al. 2024).

### Transforming lignin into high-value chemicals: strategies and advances

Lignin is increasingly investigated as a renewable substitute for phenol in synthesizing resins such as phenol-formaldehyde. However, despite its promising potential, the high cost of lignin conversion remains a major challenge, often exceeding that of conventional fossil-based processes. Recent research has identified efficient pathways for producing monomeric lignin-derived compounds such as γ-valerolactone by integrating lignin-derived intermediates like vanillin and specific alcohols into epoxy resin formulations (Hilares et al. 2020). Hydrogenolysis of hardwood lignin can yield up to 51% monomeric aromatics such as guaiacol, syringol, and vanillin and softwood lignin typically yields around 23% due to its more condensed molecular structure (Evstigneyev and Sergey 2020).

The production of lignin-based chemicals is hindered by the complexity of mixtures generated through conventional lignin degradation methods. However, recent advances have identified bacterial enzymes with lignin-degrading capabilities, offering new opportunities for renewable chemical synthesis. In addition, various strategies including non-catalytic and catalytic thermochemical processes, as well as chemo-catalytic and biocatalytic approaches, have demonstrated significant potential in overcoming these challenges (Bugg et al. 2020).

Vanillin, or 4-hydroxy-3-methoxybenzaldehyde, is a prominent monomeric phenolic compound derived from lignin, accounting for approximately 20% of such compounds in biomass. Notably, it is the only lignin-derived phenolic produced on an industrial scale, primarily through the Kraft process, with an annual production exceeding 9,000 tonnes. Vanillin, a primary component responsible for vanilla's characteristic flavor, finds extensive applications across diverse industries, including food and beverages, perfumery, and pharmaceutical synthesis. Studies have also explored vanillin’s protective effects against diabetic nephropathy, a common diabetes-related condition that affects kidney function (Zabad et al. 2019). Acidic oxidation of sulfonated lignin has been reported to produce vanillin derivatives at a maximum yield of approximately 5%. Enzymatically treated lignin has demonstrated potential for vanillin yields of up to 19%, thanks to its less condensed nature (Werhan 2013).

Coumaric acid, primarily found in nature as p-coumaric acid, is a hydroxy derivative of cinnamic acid that can be efficiently synthesized via alkaline hydrolysis. Its proven ability to reduce UV-induced cytotoxicity makes it a powerful candidate for use as an active ingredient in advanced cosmetic formulations. (Timokhin et al. 2020). Its effects on thrombogenesis and platelet activation were studied in male Sprague–Dawley rats, showing that syringic acid mitigates thrombosis and thromboembolism by inhibiting clot formation and procoagulant protease activities (Srinivasulu et al. 2018).

### Nature’s powerhouse: the biofuels and bioenergy frontier

Lignocellulosic biomass offers a promising renewable resource for biofuel production, as the depletion of fossil fuel reserves and growing environmental concerns drive the global transition toward sustainable energy alternatives (Lu and Gu 2022). Approximately 98% of lignin produced globally is utilized as a fuel source for generating heat and electricity. The calorific value of dry lignin is about 25 MJ/kg, which is comparable to coal, whose energy density typically ranges between 24 and 30 MJ/kg. Firing lignin with coal, particularly in pulping boilers, is a common practice. The study reported that this co-firing approach enhances boiler efficiency by 38% compared to systems that rely solely on black coal, while also achieving a 60% reduction in carbon emissions (Bajwa et al. 2019). The production of biophenols and biopolyols from lignin has also gained traction (Dessbesell et al. 2020), reflecting the global kraft lignin market growth from USD 1.27 billion in 2023 to USD 1.34 billion in 2024 (Kraft Lignin Market Valuation. 2024). Furthermore, the utilization of lignin as a feedstock for the production of benzene, toluene, and xylene can create new market opportunities and economic value.

Lignin can be a cost-effective raw material for carbon fibre production, providing a renewable alternative to petroleum-based sources. By using lignin-derived carbon fibres, which are less vulnerable to fluctuations in oil prices, industries can manufacture lightweight, high-strength panels for vehicles. Replacing traditional steel with these carbon fibers not only lowers production costs but also reduces vehicle weight, leading to improved fuel efficiency and overall transportation economy (Hiremath et al. 2020).

**Fig. 1:**
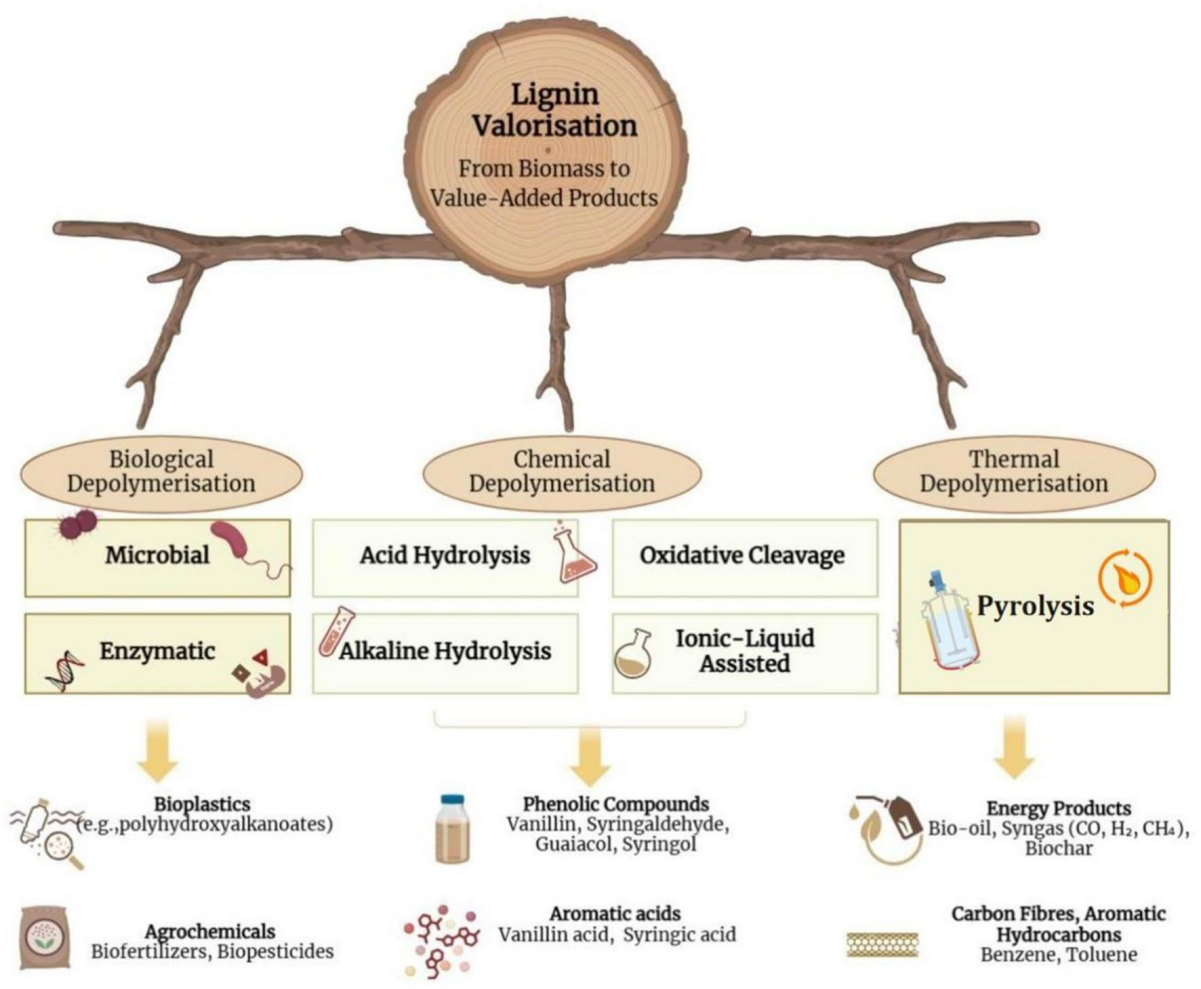
Overview of lignin valorization pathways for converting lignin into valuable chemicals, materials and fuels

Figure 1 provides an overview of lignin valorization, illustrating the key processes and pathways involved in converting lignin into valuable chemicals, fuels, and materials. This emphasizes the potential of lignin as a renewable feedstock in the development of sustainable biorefineries.

### Transforming possibilities: cutting-edge advances and innovations

The growing interest in lignin applications is largely driven by its inherent antioxidant, antimicrobial, and UV-protective characteristics. Coupled with its increasing global availability and compatibility with polymers makes lignin as an attractive, eco-friendly option for companies seeking to develop profitable and sustainable products (Bajwa et al. 2019). Lignin holds significant promise in the biomedical sector, especially in areas such as tissue engineering, wound healing, and drug delivery systems. Its suitability for these applications largely stems from its natural antimicrobial and antioxidant properties, which help inhibit bacterial growth and reduce oxidative stress (Domínguez-Robles et al. 2020).

In recent years, lignin-based hydrogels have gained attention for a variety of applications, with particular emphasis on biomedical uses such as tissue engineering, wound dressings, and drug delivery systems (Mohammadinejad et al. 2019). Lignin has shown great potential in creating advanced delivery systems, with the sustained release of hydrophobic compounds like curcumin over a span of four days. In another innovative study, lignin was combined with xanthan to form hydrogels capable of controlled bisoprolol release, offering a promising approach for the targeted treatment of hypertension and heart failure. These developments highlight lignin's versatility in drug delivery, enhancing therapeutic efficacy while promoting sustainability. (Domínguez-Robles et al. 2020). Lignin has been effectively integrated with various polymers such as chitosan, PVA, alginate, and cellulose to create hydrogels designed for wound healing and tissue engineering applications. Its incorporation in hydrogel-based wound dressings can contribute to infection prevention and promote the healing process (Zhang et al. 2019a, b; Ravishankar et al. 2019). Lignin is also utilized in the creation of nanomaterials, which significantly alter characteristics compared to their larger-scale counterparts. Lignin nanoparticles (LNPs), in particular, demonstrate enhanced UV-blocking and antioxidant capabilities compared to bulk lignin and show strong potential for applications in drug and gene delivery systems (Zhang et al. 2019a, b; Domínguez-Robles et al. 2020). Recent studies have explored the potential of lignin as a pharmaceutical excipient to improve drug bioavailability. One study demonstrated that incorporating lignin with other excipients enhanced the release rate of aspirin from tablets, while another investigation showed that combining lignin with microcrystalline cellulose (MCC) modified the dissolution profile of tablets. These findings underscore lignin's promising role in optimizing the performance of oral drug formulations (Domínguez-Robles et al. 2019; Pishnamazi et al. 2019a, b).

An emerging lignin application closely linked to the biomedical field is the formulation of lignin-based cosmetics. Although typically associated with personal care and beauty, many modern cosmetic products are now designed to address mild skin conditions, including acne and atopic skin (Majtan et al. 2021). Lignin has gained considerable attention for its antioxidant and UV-blocking properties. Its radical scavenging ability is primarily attributed to the presence of free phenolic hydroxyl groups and ortho-methoxyl substitutions on its aromatic rings. Additionally, its UV absorption capacity is due to chromophoric groups such as phenolics, hydroxyls, double bonds, and carbonyls within its structure. Utilizing lignin as a sun-blocking agent offers distinct advantages, including enhanced SPF performance in formulations and improved photostability of conventional UV filters (Antunes et al. 2023). Lignin's potential as a pharmaceutical excipient offers opportunities for a range of applications and should be further explored. Additionally, studies have indicated that incorporating lignin into commercial sunscreen formulations can enhance their sun protection factor (SPF) (Lee et al. 2020).

Real-time monitoring through wearable sensors has become a rapidly growing field, with research teams worldwide actively developing innovative materials for these devices (Collins et al. 2022). Hydrogels are three-dimensional, cross-linked polymer networks capable of retaining large amounts of water. Their inherent adhesive properties and the ability to incorporate conductive materials into the polymer matrix make them ideal for use in wearable sensor technologies. In one study, etherified alkali lignin was utilized to develop biocompatible allyloxy-hydroxypropyl (AHP)/lignin/polyacrylic acid (PAA) hydrogels, which demonstrated strong UV-shielding stability and effective antioxidant properties for neutralizing free radicals. These hydrogels also showed high sensitivity to pressure, making them suitable for detecting a range of pressure variations (Wang et al. 2021).

**Fig. 2:**
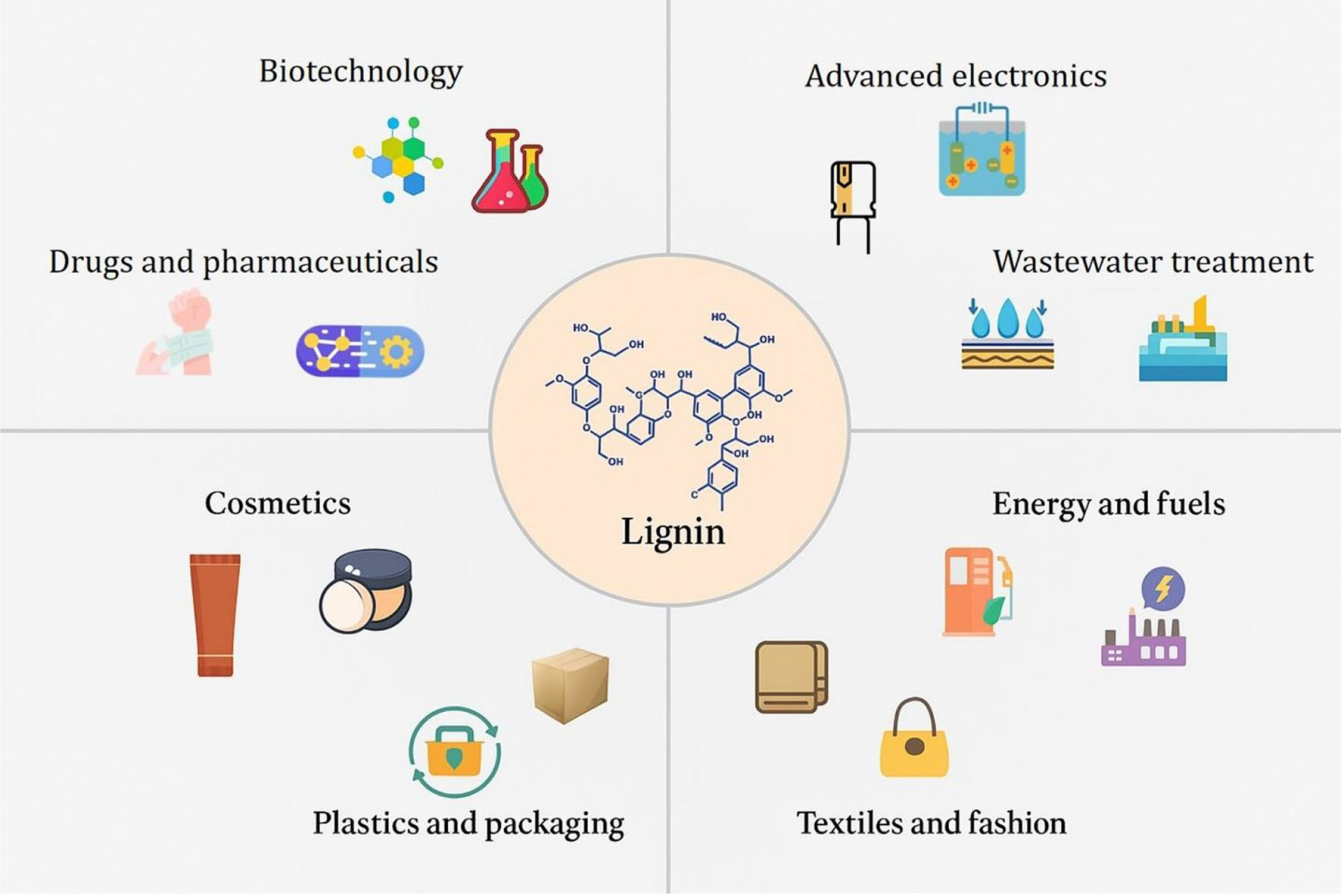
Lignin transformation and its applications in various industrial sectors

Recent advancements have seen the integration of lignin with quantum dots. In a study by Paul et al. (Paul et al. 2022), lignin-cadmium sulfide quantum dots were developed, demonstrating the ability to detect copper and mercury ions in water samples. Another notable use of lignin-based materials in wastewater treatment is their ability to remove heavy metals like cadmium, zinc, and lead. To improve lignin's effectiveness in adsorbing these metals, it typically requires chemical and physical modifications. Heavy metals pose serious health risks to both humans and animals and can be lethal when found in high concentrations (Barman et al. 2022). These advancements in lignin-based technologies underscore lignin's immense potential as a sustainable, multifunctional material driving innovation across diverse scientific and industrial domains. Figure 2 illustrates the transformation of lignin across various industries.

### Lignin valorization: a green pathway to economic and environmental benefits

Lignin valorization supports sustainability by promoting a circular bioeconomy, lowering industrial carbon footprints, and improving resource efficiency through integrated biorefineries. Life cycle analysis (LCA) shows that lignin valorization uses less water and energy while reducing environmental impact. Recent advances improve its technoeconomic feasibility, enabling its use in high-value, eco-friendly alternatives to petrochemical products (Ali et al. 2024). Integrating lignin into biorefinery processes maximizes biomass utilization and supports zero-waste principles, effectively addressing key challenges in waste management and environmental conservation. (Dessie et al. 2023; Bilal et al. 2024). Studies have highlighted the role of lignin in biodegradable composites, showing enhanced mechanical properties when combined with other bio-based polymers. Further, LCA studies have shown that lignin-based composites exhibit reduced ecological footprints across their life cycle, reinforcing their role as sustainable material solutions (Vasile and Baican 2023).

The production of valuable byproducts such as biochar, syngas, and biofuel reduces greenhouse gas (GHG) emissions over its life cycle. Technoeconomic assessments reveal that biochar enhances carbon sequestration and soil health, while syngas and biofuels offer renewable alternatives, cutting emissions by over 60% compared to conventional fossil fuels (Baral et al. 2024; Mandal et al. 2022). Additionally, case studies illustrate the potential of lignin-derived materials in antimicrobial applications, lowering the carbon footprint of the industry, expanding their applications in packaging and biomedical fields (Chen et al. 2023). Real-world case studies on lignin-derived biofuels highlight successful commercialization efforts, particularly in pilot-scale biorefineries that enhance lignin fractionation processes for optimized energy production (Brienza et al. 2024).

**Fig. 3:**
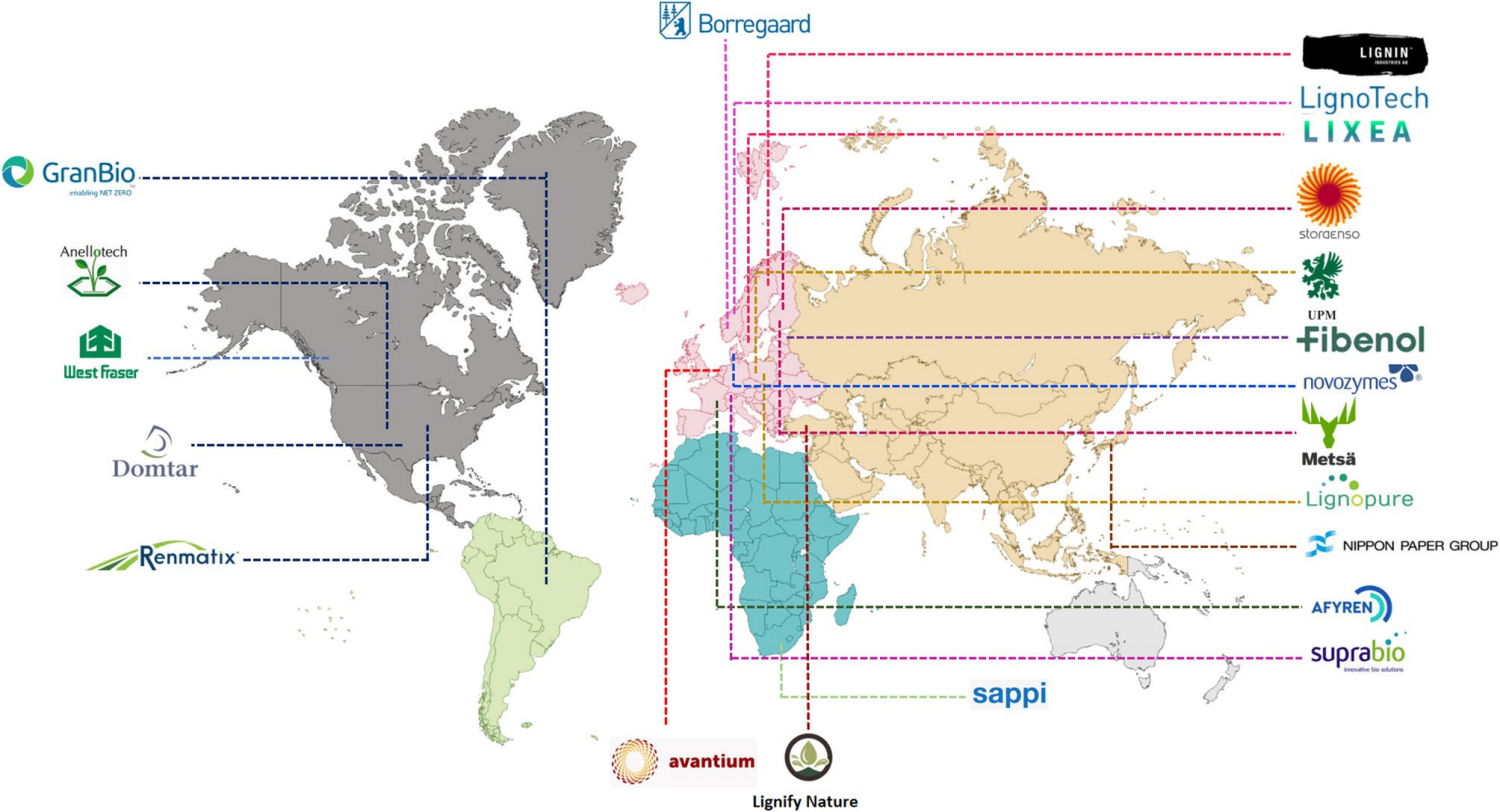
The global distribution of lignin-based industries

Economically, lignin valorization presents a promising avenue for cost-effective material production. The integration of lignin into adhesives, coatings, and bioplastics has shown potential in reducing manufacturing costs while maintaining high-performance characteristics. The economic feasibility of lignin-based materials is further reinforced by advancements in fractionation methods, which enable selective extraction of high-purity lignin suitable for specialized applications (Vasile and Baican 2023). Life cycle cost assessments highlight softwood-derived 2-pyrone-4,6-dicarboxylic acid (PDC) as a leading revenue generator, contributing to renewable high-value products (Pérez-Boada et al. 2005). Furthermore, technoeconomic case studies emphasize the growing demand for lignin-derived vanillin, syringaldehyde, and biophenols, reinforcing the global market's increasing traction (Dessbesell et al. 2020). According to market projections, the global kraft lignin sector has expanded from USD 1.27 billion in 2023 to USD 1.34 billion in 2024, reflecting a steady upward trajectory (Lignin Market Size and Outlook 2025). As countries worldwide advance lignin-based technologies, Fig. 3 highlights the growing momentum of lignin valorization by showcasing the global distribution of lignin-focused industries.

Lignin is emerging as a cost-effective precursor for carbon fiber production, especially in the automotive industry. Its use in lightweight, high-strength components reduces reliance on petroleum-based materials. Life cycle assessments indicate that lignin-derived carbon fibers improve fuel efficiency by reducing vehicle weight, resulting in lower emissions and long-term cost savings (Hiremath et al. 2020). Research shows that lignin-based carbon fiber-reinforced plastics (CFRP) reduce both production costs and CO₂ emissions, offering environmental and economic benefits. Their adoption in the aerospace and automotive industries is growing due to less energy-intensive processing compared to traditional carbon fibers (Mandal et al. 2022). The use of lignin in bio-leather and sustainable textiles is gaining traction, offering industries an alternative to synthetic materials while promoting circular economy principles. The development of lignin-derived nanoparticles and hydrogels has further expanded its market potential, enabling applications in drug delivery systems and environmental remediation (Wenger et al. 2020). Emerging research suggests that lignin's antioxidant and UV-blocking properties could be leveraged in protective coatings and cosmetic formulations, further increasing its commercial viability (Vasile and Baican 2023). Successful commercialization of lignin-based technologies requires collaboration across materials science, engineering, and market analysis (Wei et al. 2024). As research continues to refine lignin utilization strategies, its role in sustainable material development is expected to grow, contributing to both environmental conservation and economic progress. The industry leaders, such as Borregaard, Domtar, Stora Enso, and Nippon Paper, among others, commercialize different lignin-based products by using different valorization techniques Table 3.

**Table 3:** Current lignin valorization techniques and products of leading global companies

Company	Country	Method of valorization /Lignin source	Products	References
Stora Enso	Finland	Lignin extraction from wood pulp	Lignin-based resins, adhesives, carbon materials	Stora Enso 2025
Borregaard	Norway	Lignin biorefinery processes	Vanillin, lignosulfonates, bioplastics	Borregaard 2025
Domtar	USA	Kraft lignin recovery	Lignin-based dispersants, binders, carbon fibers	Domtar 2025
Renmatix	USA	Supercritical hydrolysis	Bio-based chemicals, lignin derivatives	Renmatix 2025
AVAPCO	USA	Acid hydrolysis technology	Cellulosic ethanol, lignin coproducts	AVAP 2022
West Fraser	Canada	Lignin extraction from kraft pulping	Lignin-based additives, adhesives	West Fraser 2025
Nippon Paper Industries	Japan	Lignin separation via kraft pulping	Functional lignin derivatives	Nippon Paper Group 2025
Anellotech	USA	Thermal catalytic lignin depolymerization	Bio-based aromatic chemicals	Anellotech 2008
Fibenol	Estonia	Biorefinery using hydrothermal treatment	High-purity lignin, biofuels	Fibenol 2025
LignoTech	Norway	Lignin-based chemical production	Lignosulfonates, vanillin	Borregaard 2025
Sappi	South Africa	Lignin recovery from kraft pulping	Lignin-based dispersants, emulsifiers	Sappi Global 2025
UPM Biochemicals	Germany	Fractionation of lignin	Lignin-based resins, coatings	UPM Biochemicals 2025
Lignin Industries	Sweden	Mechanical and chemical fractionation	Bio-based plastics, adhesives	Lignin Industries 2024
GreenValue SA	Switzerland	Lignosulfonate production	Dispersants, surfactants	GreenValue SA 2025
Metsa Group	Finland	Lignin extraction during pulping	Lignin-based adhesives, carbon materials	Metsä Group 2025
Novozymes	Denmark	Enzymatic valorization of lignin	Enzymatic lignin for biofuels, biochemicals	Novozymes 2025
Afyren	France	Biotechnological valorization	Bio-based organic acids, biopolymers	Afyren 2024
Avantium	Netherlands	Catalytic lignin conversion	Bio-based aromatics, chemicals	Avantium 2025
Lixea	Sweden	Dendrinic process ionic liquid extraction	Lignin, vanillin, materials and fuels	Lixea 2025
Lignify	Turkey	Lignin-based bio-material production	Vegan leather	Lignify 2025
LignoPure GmbH	Germany	Extraction, purification, and functionalization	Cosmetics, microplastic, nutraceuticals, leather	Lignopure 2025

### Hurdles and hopes in lignin valorization and product innovation

Despite its potential, lignin commercialization faces challenges in ensuring consistent quality across biomass sources. Advances in fractionation and chemical modification are helping overcome these issues, making lignin-based biomaterials more viable for industrial use (Meraj et al. 2023). The primary challenge in lignin valorization lies in its structural complexity and heterogeneity, which hinder efficient processing and product consistency (Ullah et al. 2022; Wang et al. 2019a, b). The heterogeneous nature complicates its analysis and characterization, rendering standard analytical methods insufficient for comprehensive evaluation. This complexity necessitates the development of advanced analytical techniques to better identify effective pathways and products (Ali et al. 2024). The extraction processes, such as the Kraft process, are not only energy-intensive but also contribute to material losses and raise environmental concerns, further complicating lignin valorization efforts (Sapouna et al. 2023; Beaucamp et al. 2022). The recovery of valuable aromatic compounds from lignin depolymerization is hindered by technically demanding separation and purification processes, as the similar physical and chemical properties of lignin-derived products demand labor-intensive methods and the use of hazardous solvents, leading to significant inefficiencies (Ali et al. 2024). The three-dimensional structure and limited results in stable configurations hinder efficient depolymerization, often leading to low yields and selectivity in product formation (Tian et al. 2023). Continued research in lignin structure analysis and conversion technologies is essential for producing tailored chemicals and fuels from this renewable resource (Cao et al. 2019).

Thermal depolymerization, demands high energy input and offers limited selectivity, resulting in byproducts like char and tar complicate product separation (Zhou et al. 2022). Catalytic depolymerization offers selective product formation and holds promise for early commercial applications like biorefineries. However, its scalability is limited by high catalyst costs, deactivation, complex operating conditions, and ongoing challenges in catalyst recovery, recycling, and overall cost efficiency (Rana et al. 2023; Biswas et al. 2021). Biological methods, mainly using microbial or enzymatic systems, are limited by slow reaction rates and sensitivity to operating conditions (Gu et al. 2024). Furthermore, oxidative depolymerization methods provide effective lignin bond cleavage but struggle with high oxidant demands, potential environmental concerns, and unwanted byproducts (Abdelaziz et al. 2022; Junghans et al. 2020; Pang 2019). Reductive depolymerization offers selective bond cleavage but faces challenges with costly and complex reaction conditions and inefficient recovery and reuse of reducing agents (Huang et al. 2024). To make this process viable in future biorefinery systems, ongoing research is essential to improve efficiency, selectivity, cost-effectiveness, and environmental sustainability. Catalytic approaches, in particular, offer the most immediate industrial promise, and with continued advancements, they are well positioned to enable a more sustainable and scalable path for lignin valorization.

Lignin valorization faces economic challenges due to high processing costs involving energy, catalysts, and specialized equipment, making its products less competitive than fossil-based alternatives (Wang et al. 2019a, b). The lack of established markets further limits demand and investment (Sivagurunathan et al. 2021; Wang et al. 2019a, b). Scaling up from pilot to industrial levels presents technical barriers, especially in thermal, catalytic, and biological methods, which often suffer from limited heat transfer and high operational expenses (Ali et al. 2024; Roy et al. 2022). Additional hurdles include regulatory compliance and inefficient byproduct recovery, highlighting the need for enhanced process efficiency, sustainable feedstock use, and better market integration (Ali et al. 2024; Zhou 2020; Syed et al. 2024).

A major challenge lies in the incorporation of lignin is the inherent incompatibility between the typically linear aliphatic structure of biodegradable polymers and the complex, aromatic, and cross-linked nature of lignin. This mismatch often results in phase separation and poor material homogeneity, which compromises the performance of the final product. Similar issues arise in the development of nanocomposites, where incorporating higher amounts of lignin nanoparticles is hindered by particle aggregation and dispersion difficulties. Additionally, concerns about the interaction of lignin-based materials with their environment whether in contact with food, living tissue, or soil, remain underexplored, and comprehensive safety assessments, including *in vivo* evaluations, are necessary for broader application (Boarino and Klok 2023). The processing and application of lignin as a functional biomaterial continue to face significant obstacles. Most of the lignin currently available is derived from the pulp and paper industry, where intensive mechanical and chemical treatments introduce various impurities, including salts and harmful substances. As a result, a readily usable and uncontaminated lignin source for biomaterial applications is lacking. Among the promising alternatives is organosolv lignin, which is extracted using environmentally friendly organic solvents like methanol or acetone. This method not only minimizes ecological impact but also yields a purer form of lignin compared to conventional extraction techniques (Sugiarto et al. 2022).

Developing bio-based leather using lignin, through innovative and eco-friendly methods, often demands significant research investment, advanced technology, and specialized infrastructure, which collectively lead to higher production costs compared to traditional leather. Overcoming this barrier requires continuous efforts to reduce costs, improve production scalability, and enhance manufacturing efficiency (Lignin Market Size and Outlook 2025). In terms of durability, many bioleather products may not match the lifespan of premium animal leather, often showing signs of wear within 3–5 years. This makes them less suitable for demanding applications such as industrial footwear or automotive interiors. Additionally, limited large-scale production results in higher market prices and restricted design variety when compared to conventional leather. A further concern is that biodegradability is not always assured only fully plant-based formulations without synthetic polymers like polyurethane tend to break down completely. Many commercial versions of bio-leather are not suitable for home composting, posing sustainability challenges (Gionar 2015).

### Strategic outlook and emerging directions

Lignin, one of the most abundant yet underutilized components of lignocellulosic biomass, holds tremendous potential in advancing a sustainable bioeconomy. As industries shift toward renewable resources, lignin's valorization into high-performance biomaterials, biofuels, and value-added chemicals can significantly reduce dependence on fossil-derived products. These innovations directly contribute to SDG 12 (Responsible Consumption and Production) by promoting resource efficiency and waste reduction, and to SDG 13 (Climate Action) by lowering carbon emissions. Harnessing lignin's full potential is key to accelerating the transition to circular, low-carbon industries aligned with global sustainability goals.

One of the most promising research directions involves precision depolymerization using tailored catalysts for both chemical and enzymatic processes. Biocatalytic systems, including enzymes like lignin peroxidase, versatile peroxidase, and engineered laccases, have shown potential for breaking down lignin into specific aromatic monomers under milder and more environmentally friendly conditions. However, despite these advancements, significant technological bottlenecks persist. A critical challenge is the limited solubility of lignin due to its heterogeneous and recalcitrant structure. This structural complexity, arising from irregular bonding patterns and high molecular weight fractions, hinders uniform depolymerization and downstream processing. Current methods often struggle to selectively solubilize or fractionate lignin based on molecular weight, resulting in inconsistent yields and limited product quality. Addressing these limitations requires the development of advanced solvent systems, fractionation techniques, and standard protocols to tailor lignin solubility and functionality for specific applications. Moving forward, advancing lignin valorization will require high-throughput catalyst discovery, detailed structural profiling, and AI-assisted computational modeling to optimize reaction conditions, guide catalyst design, and enable real-time monitoring. These innovations aim to reduce experimental cycles and support scalable, cost-effective processing.

The molecular weight of lignin is a critical factor influencing its suitability for various biomaterial applications. Lower molecular weight fractions tend to exhibit higher reactivity, making them ideal for the production of fine chemicals, adhesives, and resins. In contrast, higher molecular weight fractions are better suited for structural biopolymers and composite materials. Therefore, future lignin valorization strategies must emphasize precise control of molecular weight distribution and its alignment with specific end-use performance requirements.

The integration of lignin into bio-based composite materials is gaining significant momentum. Its inherent properties such as UV resistance, thermal stability, and antimicrobial activity make it a strong candidate for replacing petroleum-derived polymers in sectors like textiles, automotive, aerospace, and construction. Researchers are developing lignin-reinforced thermoplastics, thermosetting resins, and even carbon fiber precursors, offering lightweight, durable, and eco-friendly alternatives. In particular, lignin nanoparticles (LNPs) represent a cutting-edge innovation, offering benefits such as high antioxidant activity, enhanced compatibility in polymer matrices, and tunable surface chemistry. These characteristics make LNPs ideal for diverse applications, ranging from drug delivery and cosmetics to strengthening biodegradable plastics.

Beyond structural materials, lignin also holds significant potential in environmental and energy-related applications. Its conversion into activated carbon provides a sustainable route for carbon dioxide capture, water purification, and soil amendment through biochar production. Moreover, lignin-derived carbon materials are being explored for use in green energy storage devices such as supercapacitors and batteries. These lignin-based electrodes offer a renewable alternative to traditional materials in lithium-ion and sodium-ion battery systems, aligning with the push for cleaner energy solutions.

In the European Union (EU), lignin valorization has been integrated into its bioeconomy strategy and circular economy action plan, which promote the use of renewable biomass to decrease dependency on fossil-based products (European Commission 2025). Similarly, carbon pricing mechanisms, such as the EU Emissions Trading System (EU ETS), indirectly benefit lignin-based solutions by raising the cost of fossil-derived carbon and creating economic incentives for companies to adopt bio-based alternatives (European Environment Agency 2025). Additionally, many countries now provide targeted funding to boost lignin valorization technologies. For instance, the United States Department of Energy- Bioenergy Technologies Office (BETO) allocates grants to advance lignin research, encouraging the development of high-value products derived from lignin for a thriving bioeconomy (Bio Energy 2025). Embracing lignin valorization stands as a pivotal opportunity to transform industries and pave the way for a resilient, sustainable bioeconomy, where nature's own materials replace fossil-derived resources and contribute to a cleaner, greener world.

To realize the full potential of lignin valorization and its application, interdisciplinary innovative collaborations are crucial. Partnerships among research institutions, industries, and policymakers can help overcome technological barriers, standardize processing methods, and establish economically viable supply chains. Policy support in the form of research grants, tax incentives, and sustainability-linked regulations is essential to accelerate innovation and commercial adoption of lignin-based technologies.

## Conclusions

Lignin valorization is emerging as a key strategy for a sustainable bioeconomy, offering significant potential to replace fossil-based products across various sectors. As a major component of lignocellulosic biomass, lignin can be converted into high-value chemicals, biofuels, bioplastics, and advanced materials. It plays a crucial role in supporting a circular economy, despite challenges such as scalability and processing costs. Global efforts focused on bioeconomy and low-carbon transitions are driving research in lignin valorization, leveraging its multifunctionality to reduce carbon emissions and improve resource efficiency. Among lignin valorization methods, chemical depolymerization is noteworthy for producing valuable phenolic monomers and oligomers that can be used in bioplastics, adhesives, and fine chemicals. When combined with thermochemical processes like pyrolysis, it enhances renewable fuel production. Microbial valorization, involving enzymatic breakdown by fungi and bacteria, offers a sustainable route to bio-based phenolics and biofuels. Compared to harsh thermal depolymerization methods and the relatively inefficient biological approaches, chemical depolymerization of lignin offers improved reaction control and higher product selectivity, making it a more effective strategy for converting lignin into renewable fuels and valuable chemicals. So, Integrating chemical, thermochemical, and microbial techniques maximizes lignin's potential.

Lignin's structural and functional properties make it an ideal material for use in bioplastics, packaging, biomedical devices, and flame-retardant systems. It can replace polyols in polyurethanes, improving elasticity, and has emerging applications in conductive gels, carbon fibers, and batteries. Its derivatives, like vanillin, are commercially successful, and other phenolics offer UV protection and therapeutic benefits. Despite its potential, challenges remain in scaling lignin-based products due to complex extraction processes, cost inefficiencies, and material inconsistency. However, advancements in green chemistry and process optimization are improving its viability. As global climate action accelerates, lignin's valorization supports the transition to low-carbon economies, offering both economic opportunities and environmental benefits in the pursuit of a sustainable, circular global economy.

## Supplementary Information

Below is the link to the electronic supplementary material.

Supplementary Material 1

## Data Availability

Not applicable.
