# Supplementary figures and images for: Sustainability through lignin valorization: recent innovations and applications driving industrial transformation
Source: Bioresour Bioprocess. 2025 Aug 22;12(1):88. doi: 10.1186/s40643-025-00929-x (PMC12373608; doi:10.1186/s40643-025-00929-x)

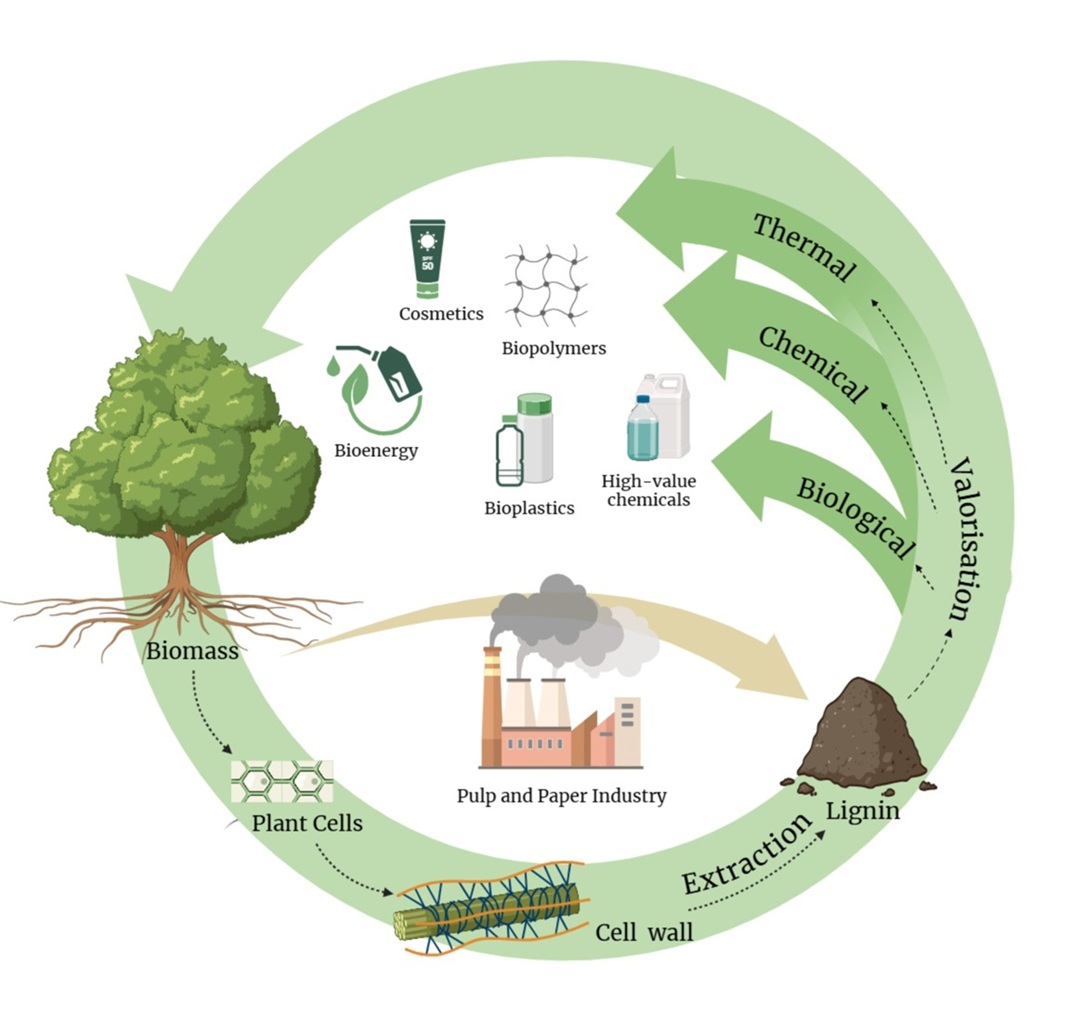

Supplement: Supplementary file 1 — Supplementary Material 1 [file 40643_2025_929_MOESM1_ESM.jpg]
